# Association of Expanded Child Tax Credit Payments With Child Abuse and Neglect Emergency Department Visits

**DOI:** 10.1001/jamanetworkopen.2022.55639

**Published:** 2023-02-16

**Authors:** Lindsey Rose Bullinger, Angela Boy

**Affiliations:** 1 School of Public Policy, Georgia Institute of Technology, Atlanta; 2 Stephanie Blank Center for Safe and Healthy Children, Children’s Healthcare of Atlanta, Atlanta, Georgia

## Abstract

**Importance:**

Poverty has long been associated with a higher risk for child maltreatment, and recent research suggests that income support policies are associated with reduced child abuse and neglect. However, income supports tied to employment cannot separate the associations of income from those of employment.

**Objective:**

To estimate the short-term association of universal and unconditional income payments to parents with child abuse and neglect.

**Design, Setting, and Participants:**

In this cross-sectional study, variation in the timing of the 2021 expanded child tax credit (CTC) advance payments was used to determine whether receiving unconditional income is associated with child abuse and neglect. A fixed-effects approach was used to compare child abuse and neglect before and after payments were made in 2021. The study compared 2021 trends with the same period in 2018 and 2019, during which no CTC payments were made. Participants were pediatric emergency department (ED) patients identified as experiencing child abuse or neglect at a level I pediatric hospital system in the Southeastern US from July through December 2021. Data were analyzed from July to August 2022.

**Exposures:**

Timing in the disbursement of the expanded CTC advance payments.

**Main Outcomes and Measures:**

Daily child abuse and neglect-related ED visits.

**Results:**

During the study period, there were 3169 ED visits related to child abuse or neglect. The 2021 advance payments of the expanded CTC were associated with fewer child abuse and neglect-related ED visits. There was a decrease in these ED visits in the 4 days following the advance CTC payments, although the reduction was not significant (point estimate, −0.22; 95% CI, −0.45 to 0.01; *P* = .06). There were significant reductions in such ED visits among male children (point estimate, −0.40; 95% CI, −0.75 to −0.06; *P* = .02) and non-Hispanic White children (point estimate, −0.69; 95% CI, −1.22 to −0.17; *P* = .01). These reductions did not persist, however.

**Conclusions and Relevance:**

These findings suggest that federal income supports to parents are associated with immediate reductions in child abuse and neglect-related ED visits. These results are important for discussions of making the temporary expansion of the CTC permanent and are applicable to income support policies more broadly.

## Key Points

**Question:**

Was the 2021 expanded child tax credit associated with child abuse and neglect-related emergency department (ED) visits?

**Findings:**

This cross-sectional study of 3169 ED visits related to child abuse or neglect found fewer child abuse and neglect-related ED visits in the 4 days following expanded child tax credit payments. These declines did not persist.

**Meaning:**

These findings suggest that federal income supports to parents are associated with immediate reductions in child abuse and neglect-related ED visits.

## Teaser

This cross-sectional study evaluates the association between the 2021 expanded child tax credit payments and child abuse and neglect emergency department (ED) visits.

## Introduction

Approximately 37% of all US children are the subject of an investigation by child protective services during childhood,^1^ and children in low-income families are at a substantially higher rate of maltreatment than children in higher-income households.^2^ A growing body of research supports the idea that one of the most effective means of preventing child maltreatment—particularly neglect—is by providing income support to families. For example, a more generous Earned Income Tax Credit (EITC) and higher minimum wages have been shown to reduce child maltreatment and foster care entry.^3,4,5,6,7,8^ However, these studies are unable to separately estimate the effects of income and those of employment-related decisions, such as whether, how much, and when to work. These employment decisions can also affect child maltreatment, which can confound the estimated effects of income support.^8,9^

The Child Tax Credit (CTC) is a partially refundable credit to tax-filing parents that is typically delivered to families as a lump-sum payment via a tax refund. Under the American Rescue Plan Act of 2021 (referred to hereafter as the American Rescue Plan), the CTC increased by $1000 to $1600 per child and expanded eligibility to include parents of 17-year-olds.^10^ The expansion also included advanced payments—disbursed before tax refund season—of up to $300 per month for each child younger than 6 years and up to $250 per month for each child aged 6 through 17 years to eligible families.^10^ To be eligible, families had to have filed a 2019 or 2020 tax return and claimed the CTC on the return. Alternatively, families could have provided information to the Internal Revenue Service to get stimulus payments during the COVID-19 pandemic. Additionally, families must have lived in the US for more than half the year, have a child younger than 18 years at the end of 2021, and documented incomes below $150 000.

Approximately 38 million families, representing 61.9 million children, received the advanced CTC payments during the fall of 2021.^11^ Among a nationally representative sample of families with annual earnings less than $75 000 surveyed in October 2021, 77% of respondents claimed the CTC.^12^ Among those filing their taxes, Hispanic and non-Hispanic Black respondents and those with lower education were less likely to claim the CTC.^12^ In the state of Georgia—the focus of this study—more than 96% of children with a Social Security number in the tax year 2019 were claimed.^13^

According to studies^14,15,16^ using the US Census Bureau’s Household Pulse Survey, these advanced CTC payments reduced financial hardship and food insecurity. Qualitative data suggest that households used these CTC payments to meet essential needs of a household, such as housing, utilities, food, and emergency funds, and to pay for expenses such as childcare and school-related materials.^17^

Because experiencing material hardship is associated with child maltreatment,^18^ we expect these CTC payments to also be associated with child abuse and neglect (CAN) in the short run by potentially reducing material hardship, reducing parental stress, and improving family functioning. For instance, previous research^5^ has found that in the 4 weeks following disbursement of EITC and CTC refunds, child maltreatment reports declined. These refunds constitute up to 45% of a family’s annual earnings in 1 lump sum payment^19^ and are conditional on having earnings from employment. Recent research has also shown that parents increase their child-specific spending, such as clothing and school equipment, in the month following receipt of unconditional income,^20^ which suggests an important pathway for the prevention of neglect. In contrast, other research has shown that factors associated with increased risk of child maltreatment, such as domestic violence^21^ and substance use–related crimes,^22^ increase in the days following the disbursement of unconditional income, which may suggest an increase in maltreatment.

In this cross-sectional study, we leverage public policy–induced variation in the payment schedule of the expanded CTC under the American Rescue Plan to examine the association of income payments with CAN. Specifically, we focus on the 6 advanced CTC payments that were made monthly during the second half of 2021. We use medical records data to calculate daily emergency department (ED) visit data for the months of July through December, comparing trends in CAN-related ED visits relative to the disbursement of the CTC payments in 2021 with a baseline comparison, which is the same period in previous years. This analytical design allows us to isolate the association between income and child maltreatment–related ED visits in the short term.

## Methods

This study used data from patient medical record reviews from the Children’s Healthcare of Atlanta (referred to hereafter as Children’s) system from July through December of 2018, 2019, and 2021. We omit the year 2020 in the main analyses because of the documented changes in child maltreatment as a result of the COVID-19 pandemic and subsequent public policy.^23^ These differences in the composition of cases that appeared in the ED in 2020 suggest 2020 is not a sufficient baseline comparison year. This research was approved by both Children’s and Georgia Institute of Technology institutional review boards, which determined that all specified criteria described in 45 CFR §46.116(d) were met as necessary to obtain a waiver of informed consent, because the study carried no more than a minimal risk to patients. This study follows the Strengthening the Reporting of Observational Studies in Epidemiology (STROBE) reporting guideline for observational studies.

The Children’s system serves patients from all 159 Georgia counties and treats more than 500 000 children per year in over 1 million patient visits. More than 50% of this patient population is publicly insured. See eTable 1 in Supplement 1 for additional statistics comparing Children’s patients with children in the state of Georgia. We included patients if they were younger than 18 years, treated in the ED of 1 of the 3 hospitals that make up the Children’s system, and CAN was identified by a professional during an examination. Per Children’s system, child abuse includes physical abuse and sexual abuse. Neglect consists of medical neglect, failure to thrive, and pediatric injuries due to inadequate adult supervision, including injuries that were unwitnessed, ingestions (unintentional or intentional), drownings or near drownings, falls, or gunshot wounds.

Cases came from 3 sources: (1) social work referrals for abuse, (2) *International Statistical Classification of Diseases and Related Health Problems, Tenth Revision (ICD-10) *and Pediatric Emergency Care Applied Research Network (PECARN) coding in the electronic medical record (EMR), and (3) cases identified at weekly trauma rounds. First, when CAN is suspected in a patient, clinicians refer patients to social work. Social workers then assess the patient and refer the case to the state’s Division of Family and Children’s Services (DFCS) as indicated by Children’s policy and document the referral in the EMR. If a case was reported to DFCS as documented in the EMR, it was included in our data set. In other words, these data comprise a subset of all child maltreatment referrals to DFCS. Second, cases were also identified by reviewing all cases with a final diagnosis (*ICD-10*) or PECARN diagnosis of any type of CAN. All cases with abuse-related *ICD-10* or PECARN codes in the EMR are reported monthly by the ED to the child advocacy center. Finally, cases came from weekly trauma rounds that are attended by Children’s trauma clinicians. We removed duplicate cases.

We focus on ED visits related to CAN for several reasons. First, ED visit data are less subject to reporting biases than other measures of child maltreatment, such as referrals to child protective services agencies. This feature is especially important in light of changes in maltreatment reporting during the pandemic and the resulting elevated attention to the topic.^24,25,26^ Second, our ED visit data are available at the daily level, unlike most accessible sources of child maltreatment data, which is particularly relevant when studying the sensitivity of child maltreatment to disbursements in payments. Finally, child maltreatment reporting data are typically available with an extensive lag. To examine policies implemented within the past year and offer relevant and timely analysis for policy making, data need to be readily available and accessible.

### Measures

The primary outcome is the number of patients treated in the ED for reasons involving child CAN at the daily level. This measure is the raw number of pediatric ED visits where a professional suspected CAN, as measured by EMR review, regardless of the chief concern for the visit.

For each patient, we also have child demographic characteristics (age, sex, and race and ethnicity). Race and ethnicity are parent-reported but may not be accurately captured on data entry because this information does not consistently come through on an EMR (approximately 9% of records are missing race). We use these child demographic characteristics to determine heterogeneous associations, given previous research showing that more generous income support policies have greater consequences for younger children^6,7^ and that Hispanic and non-Hispanic Black parents were less likely to take up the expanded CTC than White parents.^12^

### Statistical Analysis

We performed all statistical analyses using Stata/MP statistical software version 17.0 (StataCorp LP). All hypothesis tests were 2-sided, with statistical significance of *P* < .05. Our approach uses a fixed-effects analysis that relies on variation in the timing of the CTC payment disbursement caused by the American Rescue Plan. We limit the analysis to the same period (weeks 27-52) of 2018, 2019, and 2021 owing to potential seasonality in CAN perpetration.^27^ The advanced payments of the CTC under the American Rescue Plan were disbursed to all eligible recipients 6 times in 2021: July 15, August 13, September 15, October 15, November 15, and December 15. Therefore, we compare the 12 days before and 15 days after each disbursement date in 2021 vs the same periods in both 2018 and 2019. We include year, month, and day-of-the-week fixed effects. More details on the empirical approach are in the eMethods in Supplement 1. Data were analyzed from July to August 2022.

In a supplementary analysis, we further split the analysis into 2 periods: July to September and October to December. Doing so allows us to examine the cumulative nature of the CTC payments for CAN. In other words, we assess whether the payments in October through December have a greater association with CAN than the first 3 payments, or if the first 3 payments were more impactful. We do so given the evidence on the cumulative impacts of income support policies on children.^3,4,6,7,8,28^

## Results

During the second half of 2018, 2019, and 2021, 343 178 children visited Children’s EDs; 3169 of these visits (0.92%) were identified as CAN cases. The number of CAN-related ED visits per day at Children’s facilities was higher in 2021 than in 2018 and 2019. Specifically, in 2021, there were a mean (SD) of 7.19 (2.66) CAN-related ED visits per day in the 12 days before each month’s CTC payment date. In 2018 and 2019, this number was 5.89 (2.54). Following the CTC payments in 2021, the mean (SD) number of child CAN-related ED visits was 6.82 (3.04), compared with 5.98 (2.67) in 2018 and 2019. In other words, in the years 2018 and 2019, the number of CAN-related ED visits to Children’s hospitals increased over the course of the month. In contrast, during 2021, they decreased.

The Figure dissects the pre-CTC and post-CTC period into 4-day increments. The 4-day period following CTC payments in 2021 (ie, days 0-3) had 1.13 fewer visits than the 4 days prior, whereas the baseline years had 0.65 more visits, suggesting a possible reduction in CAN-related ED visits after the CTC payments. Days 4 through 7 in 2021 appear to rebound back to pre-CTC payment levels, however, whereas these same days in 2018 and 2019 remain level.

**Figure.**
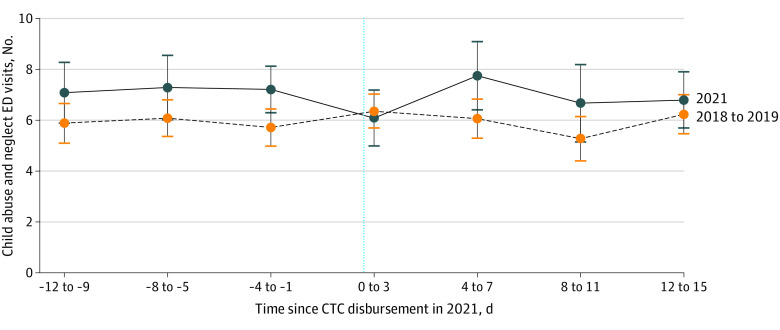
Descriptive Trends in Child Abuse and Neglect-Related Emergency Department (ED) Visits Relative to Child Tax Credit (CTC) Payment Dates in 2021 Data were obtained from Children’s Healthcare of Atlanta medical records July to December, 2018 to 2019 and 2021. The error bars represent the 95% CIs. The vertical dotted line represents the point at which CTC payments were distributed in 2021, specific to each month.

Regression results from the full model in Table 1 indicate a decrease in CAN-related ED visits in the 4 days following CTC advance payment disbursement, although the reduction was not significant (point estimate, −0.22; 95% CI, −0.45 to 0.01; *P* = .06). Relative to the baseline mean of 5.93, this reduction implies approximately 1.3 fewer visits per day. This decrease did not persist, however, because the estimates for days 4 through 15 are statistically no different from zero and smaller.

**Table 1.** Association of Advance Child Tax Credit Payments With CAN-Related ED Visits by Child Age^a^

Period	Total		Age 0-1 y		Age 2-5 y		Age 6-10 y		Age 11-17 y	
	Point estimate (95% CI)	*P* value	Point estimate (95% CI)	*P* value	Point estimate (95% CI)	*P* value	Point estimate (95% CI)	*P* value	Point estimate (95% CI)	*P* value
Days 0-3	−0.22 (−0.45 to 0.01)	.06	−0.18 (−0.62 to 0.26)	.43	−0.44 (−0.95 to 0.07)	.09	−0.07 (−0.58 to 0.43)	.77	−0.15 (−0.50 to 0.21)	.42
Days 4-7	0.01 (−0.21 to 0.23)	.92	−0.22 (−0.61 to 0.16)	.25	0.01 (−0.47 to 0.49)	.98	−0.15 (−0.77 to 0.46)	.63	0.19 (−0.16 to 0.55)	.28
Days 8-11	0.07 (−0.21 to 0.34)	.64	−0.12 (−0.64 to 0.39)	.64	0.32 (−0.17 to 0.81)	.20	0.36 (−0.22 to 0.93)	.22	−0.12 (−0.50 to 0.26)	.54
Days 12-15	−0.13 (−0.35 to 0.09)	.26	−0.33 (−0.81 to 0.15)	.18	−0.12 (−0.59 to 0.35)	.61	−0.27 (−0.90 to 0.35)	.39	0.07 (−0.30 to 0.44)	.72
CAN-related ED visits in second half of month in 2018-2019, mean No.	5.98	NA	1.49	NA	1.47	NA	0.96	NA	2.06	NA

Abbreviations: CAN, child abuse and neglect; ED, emergency department; NA, not applicable.

^a^ Data were obtained from Children's Healthcare of Atlanta medical records, July 3 through December 30 of 2018, 2019, and 2021.

In the 4 days following CTC advance payment disbursement, there were fewer CAN-related ED visits among children aged 2 through 5 years, although the reduction was not significant (point estimate, −0.44; 95% CI, −0.95 to 0.07; *P* = .09). However, there were significant reductions in such visits among male children (point estimate, −0.40; 95% CI, −0.75 to −0.06; *P* = .02) and non-Hispanic White children (point estimate, −0.69; 95% CI, −1.22 to −0.17; *P* = .01) (Table 2). We include 2020 data in a sensitivity analysis and, though they are less precise, the results are robust (eTable 2 and eTable 3 in Supplement 1).

**Table 2.** Association of Advance Child Tax Credit Payments With CAN-Related ED Visits by Child Demographic Characteristics^a^

Period	Female		Male		Non-Hispanic White		Non-Hispanic Black	
	Point estimate (95% CI)	*P* value	Point estimate (95% CI)	*P* value	Point estimate (95% CI)	*P* value	Point estimate (95% CI)	*P* value
Days 0-3	−0.09 (−0.38 to 0.21)	.57	−0.40 (−0.75 to −0.06)	.02	−0.69 (−1.22 to −0.17)	.01	−0.03 (−0.31 to 0.25)	.85
Days 4-7	0.09 (−0.17 to 0.35)	.51	−0.15 (−0.53 to 0.24)	.45	−0.02 (−0.39 to 0.36)	.92	0.03 (−0.25 to 0.32)	.81
Days 8-11	0.05 (−0.31 to 0.40)	.80	0.10 (−0.28 to 0.47)	.61	0.25 (−0.24 to 0.74)	.31	−0.01 (−0.33 to 0.32)	.97
Days 12-15	−0.18 (−0.47 to 0.10)	.20	−0.05 (−0.41 to 0.32)	.81	−0.38 (−0.79 to 0.03)	.07	−0.03 (−0.35 to 0.28)	.83
CAN-related ED visits in second half of month in 2018-2019, mean No.	3.51	NA	2.47	NA	1.74	NA	3.73	NA

Abbreviations: CAN, child abuse and neglect; ED, emergency department; NA, not applicable.

^a^ Data were obtained from Children’s Healthcare of Atlanta medical records, July 3 through December 30 of 2018, 2019, and 2021. The unit of analysis is date (n = 168 days in each of 3 years = 504). Regressions include year, month, and day of the week fixed effects and estimated as a Poisson model.

For the subgroups that yielded a main association, we next split the main sample into 2 periods: (1) July through September (the first 3 CTC advance payments) and (2) October through December (the last 3 advance payments). Table 3 reports greater associations from the last 3 advance payments compared with the association of the first 3 payments. Specifically, the magnitude of the coefficient for the first 4 days following CTC payment is nearly 3 times as large in October through December as it is in July through September, although these estimates are not significantly different from one another. Of note, December payments were made to approximately 2% more families than July payments. In October through December, the CTC advance payments were associated with a reduction in CAN-related ED visits (point estimate, −0.33; 95% CI, −0.65 to −0.01; *P* = .046). Table 3 also demonstrates greater associations in October through December among children aged 2 through 5 years (point estimate, −0.74; 95% CI, −1.44 to −0.04; *P* = .04) and non-Hispanic White children (point estimate, −1.30; 95% CI, −2.24 to −0.36; *P* = .007).

**Table 3.** Association of Advance Child Tax Credit Payments With CAN-Related ED Visits by Season^a^

Period	Total		Aged 2-5 y		Male		Non-Hispanic White	
	Point estimate (95% CI)	*P* value	Point estimate (95% CI)	*P* value	Point estimate (95% CI)	*P* value	Point estimate (95% CI)	*P* value
July through September								
Days 0-3	−0.12 (−0.44 to 0.20)	.47	−0.14. (−0.84 to 0.56)	.70	−0.47 (−0.98 to 0.05)	.07	−0.25 (−0.90 to 0.40)	.44
Days 4-7	0.19 (−0.13 to 0.51)	.24	0.49 (−0.21 to 1.20)	.17	−0.10 (−0.64 to 0.45)	.73	0.10 (−0.44 to 0.65)	.71
Days 8-11	0.16 (−0.16 to 0.49)	.33	0.78 (0.12 to 1.43)	.02	0.10 (−0.38 to 0.59)	.67	0.70 (0.06 to 1.35)	.03
Days 12-15	−0.02 (−0.32 to 0.29)	.92	0.27 (−0.35 to 0.88)	.40	0.10 (−0.39 to 0.59)	.68	−0.52 (−1.15 to 0.11)	.11
CAN-related ED visits in second half of month in 2018-2019, mean No.	6.29	NA	1.51	NA	2.56	NA	1.81	NA
October through December								
Days 0-3	−0.33 (−0.65 to −0.01)	.046	−0.74 (−1.44 to −0.04)	.04	−0.38 (−0.85 to 0.10)	.12	−1.30 (−2.24 to −0.36)	.007
Days 4-7	−0.16 (−0.45 to 0.13)	.28	−0.54 (−1.16 to 0.07)	.08	−0.18 (−0.69 to 0.33)	.49	−0.16 (−0.67 to 0.35)	.54
Days 8-11	−0.05 (−0.46 to 0.37)	.82	−0.17 (−0.85 to 0.50)	.61	0.05 (−0.49 to 0.59)	.86	−0.28 (−1.02 to 0.45)	.45
Days 12-15	−0.23 (−0.54 to 0.08)	.15	−0.59 (−1.32 to 0.14)	.12	−0.21 (−0.73 to 0.31)	.43	−0.29 (−0.82 to 0.24)	.29
CAN-related ED visits in second half of month in 2018-2019, mean No.	5.67	NA	1.44	NA	2.39	NA	1.68	NA

Abbreviations: CAN, child abuse and neglect; ED, emergency department; NA, not applicable.

^a^ Data were obtained from Children's Healthcare of Atlanta medical records, July 3 through December 30 of 2018, 2019, and 2021. The unit of analysis is date (n = 84 days in each of 3 years = 252). Regressions include year, month, and day of the week fixed effects and are estimated as a Poisson model.

## Discussion

This cross-sectional study used variation in the timing of advanced payments made through the expanded CTC to estimate the short-term associations of nearly universal and unconditional income payments on CAN. We found that in the 4 days following the advance CTC payments, CAN-related ED visits declined by approximately 22%, although this estimate was not significant, and these associations did not persist beyond these initial declines. These associations were significant among male children and non-Hispanic White children. These findings may highlight documented differences in take-up of the advanced CTC payments.

We also found that the associations of the CTC were greater from the last 3 payments than the first 3. This finding may point to the cumulative effects of the CTC payments. In other words, by October, most families had received 3 advance CTC payments already. Alternatively, it may suggest greater take-up of CTC advance payments as more payments were made, as December payments were made to approximately 2% more families than July payments.

This research adds to the evidence on the role of antipoverty policy, broadly, and tax credits, more specifically, on CAN.^3,4,5,6,28,29^ Specifically, we found that child maltreatment ED visits were sensitive to the timing of unconditional income payments. This finding is consistent with prior work showing short-term effects of tax refunds^5^ and Supplemental Nutrition Assistance Program benefit disbursement.^21^ Together, these studies suggest how child maltreatment can be influenced by even modest changes in income benefit schedules.

This study design has several strengths. First, rigorously studying the effect of income support policies on CAN is challenging owing to difficulties with randomizing income supports. Several previous studies^3,4,5,6,7,8,28,30^ have used approaches that rely on changes in public assistance programs. However, these policies often include a work requirement, such as the EITC and the minimum wage, that confound the effect of income alone. Because the CTC expansion and advance payments did not have work requirements, and empirical evidence shows no employment effects from the expanded CTC,^31^ this work-income confounding problem is alleviated.

Second, the CTC is usually delivered to families as a lump-sum payment as part of their tax refunds. Therefore, evaluating the effects of the CTC is typically a challenge because of the inability to separate the CTC from other tax refunds. The American Rescue Plan offers a unique opportunity to examine the effect of nearly universal payments to parents—they phased out only at very high incomes—over the course of several months.

Third, studies examining the role of income support policies on child maltreatment are typically unable to examine such nuanced timing. For example, the national database of child maltreatment reports is available to researchers at the biweekly level only. By having daily measures of CAN, we can leverage the exact date of CTC payment and observe how quickly income may be associated with maltreatment perpetration. Our finding of immediate associations in the days following CTC payments suggests that quick access to liquid income, not only chronic economic hardship, affects child maltreatment.

Furthermore, there were other notable policy changes affecting families with children during this period, such as the pandemic electronic benefit transfer program and the enhanced Child and Dependent Care Credit, which was also part of the American Rescue Plan. The approach we use—comparing daily trends year-over-year—alleviates concerns that these findings are confounded by these additional economic supports.

### Limitations

This study has some limitations. First, although nearly all parents received advanced CTC payments, take-up was not 100% (it was approximately 85% in Georgia).^13^ Because we do not know which parents of children visiting the ED received the CTC in each month, we are estimating intent-to-treat estimates.

This study is limited to 1 hospital system in the southeastern US. Given the consistencies of the prior work using these data with studies using data from multiple hospital systems,^23,32,33,34^ however, we expect our results are likely representative of large, urban hospital systems and their patient populations.

We focused on CAN-related ED visits instead of other measures of CAN. Child maltreatment reports typically available to researchers do not come with such fine-grained data on dates and times, and they are often available only after considerable delay. The data in this study are a subset of maltreatment reports, akin to reports seen by medical personnel, and, therefore, may not represent all child maltreatment. For example, previous research suggests that physical and sexual abuse, female children, and adolescents tend to be overrepresented in medical records data.^35^ Medical records may then omit the less obvious types of maltreatment reports that previous research suggests may be more sensitive to income support policies, such as unsubstantiated reports of neglect.^3,5,6,7^ Therefore, the results of this study are likely conservative when compared with, for example, the effects of the CTC on child maltreatment reports, but may be less conservative when compared with substantiated cases of maltreatment. Nonetheless, with 2 exceptions,^36,37^ medical records of child maltreatment cases have rarely been examined relative to economic and social policies. Prior research on the EITC,^3,4,5,6,28^ minimum wage,^7,8^ child tax credit,^5,28^ low-income housing tax credit,^30^ Supplemental Nutrition Assistance Program,^21,38^ and Medicaid^39,40^ has almost exclusively focused on child maltreatment reports, foster care admissions, or self-reported data.

Additionally, our study is limited to the first 2 weeks following the CTC payments. There may be longer-lasting effects built up over this time because of monthly payments. Estimating longer-term effects may show different patterns.

## Conclusions

This cross-sectional study examined the association of the 2021 CTC advanced payments with CAN and found immediate reductions in CAN-related ED visits in the days following disbursement of the CTC payments, which were larger after multiple payments. This study adds to the growing evidence that antipoverty policy is effective at reducing CAN. Considering the changing landscape for social and economic policy at both the state and federal levels, these findings highlight the scope of potential effects of income transfer programs.

## Notes

Supplement 1. **eTable 1. **Descriptive Statistics of Children’s Patients Relative to Children in Georgia
eMethods.
**eTable 2.** Effect of Advance CTC Payments on Child Abuse and Neglect-Related ED Visits, OLS Results **eTable 3.** Effect of Advance CTC Payments on Child Abuse and Neglect-Related ED Visits, Including 2020

Supplement 2.
Data Sharing Statement
