# Supplementary material for: Association of Expanded Child Tax Credit Payments With Child Abuse and Neglect Emergency Department Visits
Source: JAMA Netw Open. 2023 Feb 16;6(2):e2255639. doi: 10.1001/jamanetworkopen.2022.55639 (PMC9936349; doi:10.1001/jamanetworkopen.2022.55639)
Supplement: Supplement 1. — eTable 1. Descriptive Statistics of Children’s Patients Relative to Children in Georgia eMethods. eTable 2. Effect of Advance CTC Payments on Child Abuse and Neglect-Related ED Visits, OLS Results eTable 3. Effect of Advance CTC Payments on Child Abuse and Neglect-Related ED Visits, Including 2020 [file jamanetwopen-e2255639-s001.pdf]

Supplemental Online Content

Bullinger LR, Boy A. Association of expanded child tax credit payments with child abuse and neglect emergency department visits. *JAMA Netw Open*. 2023;6(2):e2255639. doi:10.1001/jamanetworkopen.2022.55639

eTable 1. Descriptive Statistics of Children's Patients Relative to Children in Georgia
eMethods.
eTable 2. Effect of Advance CTC Payments on Child Abuse and Neglect-Related ED Visits, OLS Results
eTable 3. Effect of Advance CTC Payments on Child Abuse and Neglect-Related ED Visits, Including 2020

This supplemental material has been provided by the authors to give readers additional information about their work.

© 2023 Bullinger LR et al. *JAMA Network Open.*

eTable 1. Descriptive Statistics of Children's Patients Relative to Children in Georgia

Race Data

Race	Children's Admissions	ED Visits	Georgia
American Indian/Alaska Native	0.12%	0.25%	0.67%
Asian	3.3%	2.5%	4.4%
Black/African American	39.4%	52.3%	35.4%
Pacific Islander/Hawaiian Native	0.09%	0.25%	0.22%
Other	3.2%	7.7%	4.7%
Unknown	15.4%	3.48%	NA
White	38.5%	33.6%	54.5%

Ethnicity Data

Ethnicity	Children's Admissions	ED Visits	Georgia
Hispanic	15.4%	18.5%	14.9%
Non-Hispanic	84.6%	81.3%	85.1%
Unknown	NA	0.28%	NA

Gender

Gender	Children's Admissions	ED Visits	Georgia
Male	51.5%	51.3%	50.9%
Female	48.5%	48.7%	49.1%

Payer Source

Source	Children's Admissions	Georgia
Medicaid	54.6%	39.7%
Other Public Source	6.5%	3.1%
Third-Party Payer	38.6%	50.6%
Self-Pay	2.5%	6.6%

© 2023 Bullinger LR et al. *JAMA Network Open.*

eMethods.

We estimated the following specification using Poisson estimation:

$$\log\left(E(V_{dmy}|x)\right) = \alpha + \beta_1 Day0to3_d * Year2021_y + \beta_2 Day4to7_d * Year2021_y + \beta_3 Day8to11_d * Year2021_y + \beta_4 Day12to15_d * Year2021_y + \gamma_y + \theta_m + \pi_{dow} + \varepsilon_{dmy}$$

In this model, V_{dmy} is the number of child abuse and neglect-related ED visits during date *d*, of month *m*, during year *y*. Dates span July 3 through December 30 (the second half of the year) of 2018, 2019, and 2021, totaling 540 days. The main independent variables of interest are 4 interaction variables representing 4-day increments following the CTC payment date in 2021 ($\beta_1 - \beta_4$). We adjust for (1) baseline differences in 2018 and 2019 relative to 2021 (e.g., anything related to the pandemic that is correlated with child maltreatment) by including a binary variable for each year (γ_y), (2) seasonality in trends in visits by including binary variables indicating the month of the year (θ_m), and (3) the potential differential effect of weekend days by including indicator variables for the day-of-the-week (π_{dow}). We additionally explore whether various populations were differentially affected by these payments by examining heterogeneous effects by child age (ages 0-1, 2-5, 6-10, 11-17), child sex (female, male), and child race and ethnicity (non-Hispanic white, non-Hispanic Black). Finally, due to the count nature of the variable, our main analysis is estimated using Poisson. We also estimate the model using ordinary least squares and the results are robust (see eTable 1).

The identifying assumption of this approach is that daily trends in ED visits for child abuse and neglect throughout the course of the month in 2021 would have followed similar patterns as those in 2018 and 2019. Furthermore, any correlates of child abuse and neglect ED visits are not also timed with the expanded CTC advance payments in 2021 (i.e., the 15th of the month, or the 13th of August). We believe these are reasonable assumptions given that other public programs and income supports are typically paid out in the beginning of the month or were no different in 2021 than in previous years.

© 2023 Bullinger LR et al. *JAMA Network Open.*

Sensitivity Checks

eTable 2. Effect of Advance CTC Payments on Child Abuse and Neglect-Related ED Visits, OLS Results

Effect of Advance CTC Payments on Child Abuse and Neglect-Related ED Visits, OLS Results

	Total		Aged 0-1		Aged 2-5		Aged 6-10		Aged 11-17	
	Point Estimate [95% CI]	(P-value)	Point Estimate [95% CI]	(P-value)	Point Estimate [95% CI]	(P-value)	Point Estimate [95% CI]	(P-value)	Point Estimate [95% CI]	(P-value)
Days 0-3	-1.43 [-2.89 to 0.03]	(0.055)	-0.26 [-0.94 to 0.42]	(0.454)	-0.63 [-1.35 to 0.10]	(0.089)	-0.07 [-0.61to 0.46]	(0.789)	-0.47 [-1.31to 0.37]	(0.269)
Days 4-7	0.18 [-1.41 to 1.76]	(0.828)	-0.31 [-0.89 to 0.27]	(0.290)	0.01 [-0.75 to 0.78]	(0.971)	-0.14 [-0.75 to 0.47]	(0.646)	0.62 [-0.50 to 1.73]	(0.279)
Days 8-11	0.28 [-1.49 to 2.04]	(0.759)	-0.17 [-0.85 to 0.51]	(0.628)	0.54 [-0.32 to 1.40]	(0.217)	0.31 [-0.25 to 0.87]	(0.274)	-0.41 [-1.32 to 0.50]	(0.377)
Days 12-15	-0.84 [-2.35 to 0.67]	(0.275)	-0.48 [-1.22 to 0.26]	(0.204)	-0.18 [-0.92 to 0.56]	(0.637)	-0.26 [-0.85 to 0.33]	(0.395)	0.07 [-0.91 to 1.06]	(0.885)
Mean Y in 2nd half of month in 2018-2019	5.98		1.49		1.47		0.96		2.06	

	Female		Male		Non-Hispanic White		Non-Hispanic Black	
	Point Estimate [95% CI]	(P-value)	Point Estimate [95% CI]	(P-value)	Point Estimate [95% CI]	(P-value)	Point Estimate [95% CI]	(P-value)
Days 0-3	-0.36 [-1.49 to 0.76]	(0.526)	-1.05 [-1.95 to -0.16]	(0.022)	-0.97 [-1.70 to -0.25]	(0.009)	-0.16 [-1.44 to 1.11]	(0.800)
Days 4-7	0.58 [-0.63 to 1.80]	(0.345)	-0.39 [-1.37 to 0.59]	(0.432)	0.03 [-0.67 to 0.72]	(0.939)	0.10 [-1.19 to 1.40]	(0.875)
Days 8-11	0.08 [-1.24 to 1.40]	(0.910)	0.21 [-0.79 to 1.22]	(0.679)	0.38 [-0.40 to 1.17]	(0.336)	-0.13 [-1.56 to 1.29]	(0.853)
Days 12-15	-0.68 [-1.85 to 0.49]	(0.256)	-0.15 [-1.10 to 0.81]	(0.765)	-0.66 [-1.37 to 0.06]	(0.073)	-0.20 [-1.63 to 1.22]	(0.779)
Mean Y in 2nd half of month in 2018-2019	3.51		2.47		1.74		3.73	

Source: Children's Healthcare of Atlanta data July 3 through December 30 of 2018, 2019, and 2021. Notes: The unit of analysis is date. N=168 days in each of 3 years =504. Regressions include year, month, and day of the week fixed effects and estimated using OLS.

eTable 3. Effect of Advance CTC Payments on Child Abuse and Neglect-Related ED Visits, Including 2020

	Total		Aged 0-1		Aged 2-5		Aged 6-10		Aged 11-17	
	Point Estimate [95% CI]	(P-value)	Point Estimate [95% CI]	(P-value)	Point Estimate [95% CI]	(P-value)	Point Estimate [95% CI]	(P-value)	Point Estimate [95% CI]	(P-value)
Days 0-3	-0.82 [-2.24 to 0.60]	(0.260)	-0.16 [-0.76 to 0.43]	(0.587)	-0.52 [-1.18 to 0.13]	(0.119)	-0.03 [-0.50 to 0.45]	(0.913)	-0.62 [-1.35 to 0.11]	(0.094)
Days 4-7	0.39 [-1.13 to 1.91]	(0.611)	-0.20 [-0.71 to 0.30]	(0.430)	0.01 [-0.70 to 0.72]	(0.971)	-0.10 [-0.66 to 0.45]	(0.710)	0.62 [-0.44 to 1.67]	(0.252)
Days 8-11	0.13 [-1.56 to 1.82]	(0.877)	-0.20 [-0.82 to 0.42]	(0.524)	0.52 [-0.29 to 1.33]	(0.207)	0.23 [-0.28 to 0.75]	(0.373)	-0.57 [-1.41 to 0.27]	(0.181)
Days 12-15	-0.31 [-1.74 to 1.13]	(0.675)	-0.30 [-0.96 to 0.36]	(0.378)	-0.14 [-0.79 to 0.52]	(0.681)	-0.21 [-0.74 to 0.32]	(0.444)	-0.01 [-0.92 to 0.91]	(0.991)
Mean Y in 2nd half of month in 2018-2020	6.07		0.99		0.98		0.64		1.37	

	Female		Male		Non-Hispanic White		Non-Hispanic Black	
	Point Estimate [95% CI]	(P-value)	Point Estimate [95% CI]	(P-value)	Point Estimate [95% CI]	(P-value)	Point Estimate [95% CI]	(P-value)
Days 0-3	-0.05 [-1.12 to 1.03]	(0.934)	-0.76 [-1.62 to 0.10]	(0.085)	-0.78 [-1.43 to -0.14]	(0.016)	0.06 [-1.18 to 1.30]	(0.927)
Days 4-7	0.67 [-0.52 to 1.86]	(0.267)	-0.26 [-1.18 to 0.66]	(0.575)	0.19 [-0.47 to 0.86]	(0.565)	0.11 [-1.13 to 1.35]	(0.862)
Days 8-11	0.05 [-1.21 to 1.32]	(0.932)	0.11 [-0.87 to 1.08]	(0.832)	0.38 [-0.35 to 1.10]	(0.306)	-0.31 [-1.71 to 1.08]	(0.660)
Days 12-15	-0.27 [-1.39 to 0.84]	(0.632)	-0.02 [-0.91 to 0.88]	(0.968)	-0.35 [-1.01 to 0.31]	(0.299)	-0.04 [-1.42 to 1.34]	(0.952)
Mean Y in 2nd half of month in 2018-2020	3.56		2.51		1.57		3.91	

Source: Children's Healthcare of Atlanta data July 3 through December 30 of 2018-2021. Notes: The unit of analysis is date. N=168 days in each of 4 years =672. Regressions include year, month, and day of the week fixed effects and estimated using Poisson.

© 2023 Bullinger LR et al. *JAMA Network Open.*
