# Supplementary material for: Association of Expanded Child Tax Credit Payments With Child Abuse and Neglect Emergency Department Visits
Source: JAMA Netw Open. 2023 Feb 16;6(2):e2255639. doi: 10.1001/jamanetworkopen.2022.55639 (PMC9936349; doi:10.1001/jamanetworkopen.2022.55639)
Supplement: Supplement 2. — Data Sharing Statement [file jamanetwopen-e2255639-s002.pdf]

Data Sharing Statement

Bullinger. Association of Expanded Child Tax Credit Payments With Child Abuse and Neglect Emergency Department Visits. *JAMA Netw Open*. Published February 16, 2023. doi:10.1001/jamanetworkopen.2022.55639

Data
Data available: No

Additional Information
Explanation for why data not available: The data that support the findings of this study are available from Children's Healthcare of Atlanta but restrictions apply to the availability of these data, which were used under license for the current study, and so are not publicly available.
